# Angling-Induced Barotrauma in Snapper *Chrysophrys auratus*: Are There Consequences for Reproduction?

**DOI:** 10.1371/journal.pone.0119158

**Published:** 2015-03-17

**Authors:** Laura S. Peregrin, Paul A. Butcher, Matt K. Broadhurst, Russell B. Millar

**Affiliations:** 1 Southern Cross University, National Marine Science Centre, Coffs Harbour, New South Wales, Australia; 2 NSW Department of Primary Industries, Fisheries Conservation Technology Unit, Coffs Harbour, New South Wales, Australia; 3 Department of Statistics, The University of Auckland, Auckland, New Zealand; Aristotle University of Thessaloniki, GREECE

## Abstract

In response to concerns regarding the potential for sub-lethal impacts of barotrauma on reproductively active *Chrysophrys auratus* during catch and release, 90 males and 90 females representing five reproductive stages (immature or resting-28%, developing-8%, developed-7%, ripe or spawning-23% and spent-34%) were angled from 8–70 m and macroscopically assessed (on-board and then in a laboratory). Irrespective of sex, all fish exhibited various clinical signs of barotrauma, including a prolapsed cloaca (60% of fish); gastric herniation (46%); ruptured swim bladder (73%); organ displacement (48%); and kidney (3%), liver (73%) and coloemic-cavity haemorrhaging (33%);with the frequency of nearly all positively associated with capture depth. Reproductive stage was also an important barotrauma predictor (reflecting related morphological changes) with a general trend towards spent fish least likely to incur the various clinical signs—especially for a prolapsed cloaca (also common among immature or resting fish and significantly affected by food in the digestive tract) and a ruptured swim bladder (common among ripe or spawning fish). The only macroscopically visible gonad damage was haemorrhaging, which was least common among immature or resting and spent fish and, irrespective of reproductive stage, temporally reduced in frequency, and more quickly among males than females. While further research is required to accurately describe the effects of angling at each stage of the reproductive cycle and the physiological consequences of barotrauma on the gonads of *C*. *auratus*, given the observed influences of reproductive stage and depth on barotrauma found in this study, any adverse effects might be partially managed by regulating either temporal or spatial fishing effort.

## Introduction

*Chrysophrys auratus* (‘pink snapper’) is widely distributed throughout the coastal Indo-Pacific down to depths of 200 m and like other regional sparids, is highly sought by anglers in southern Australia [1]. Despite their popularity, there are few quantitative estimates of the Australian recreational exploitation of *C*. *auratus*, with the most recent assessment (from a national creel study in 2000 and 2001) estimating a total annual harvest of ~1.3 million individuals [2]. An additional 2.5 million *C*. *auratus* were presumed released due to minimum (28–40 cm, total length, TL), and maximum (40–70 cm TL) legal size limits, daily personal quotas (1–10 fish) and also as a voluntary conservation measure [2].

The importance of *C*. *auratus* to local recreational fisheries and associated concerns over their post-release fate has resulted in several recent studies [3−8]. Short-term (< 10 days) post-release mortalities have ranged from 8-33%, with most deaths attributed to cumulative technical (e.g. hook type) and environmental (e.g. warm water temperatures) impacts, or like for several other local species notably detrimental handling such as ingested hooks [3−5,8].

While the generally low mortalities are a positive result for stocks of *C*. *auratus*, there are also concerns over sublethal impacts. In particular, one factor shown to negatively impact *C*. *auratus* is barotrauma among individuals caught from >*~*10 m [7]. Barotrauma is caused by rapid decompression and, depending on species-specific anatomical and physiological characteristics, can manifest as numerous injuries [9]. Common clinical signs of barotrauma include a distended coelomic cavity, gastric eversion, prolapsed cloaca, exophthalmia (bulging eyes), oculogyration (eye rotation) reflex, corneal or subcutaneous gas bubbles, internal organ displacement, swim-bladder rupture, and haemorrhaging [9,10]. These injuries were observed for *C*. *auratus* in two recent studies by Butcher et al. [7] and McLennan et al. [8], and although immediate mortalities were low, concerns were raised by Butcher et al. [7] over the potential impacts of morphological injuries to reproductively active individuals; which were not assessed because the studied specimens were in a resting or immature stage (for stage definitions see [1,11]).

*Chrysophrys auratus* becomes reproductively active in response to water temperatures and more specifically when the ocean surface is >18°C [11]. This typically occurs between July (winter) and November (spring) off south eastern Australia [12]. Previous studies with wild and captive *C*. *auratus* found that catching fish during spawning can negatively affect their subsequent gonadal development [13,14]. Because gonads can occupy most of the coelomic cavity in fully developed, spawning *C*. *auratus*, angling-related impacts might be exacerbated by barotrauma. Such impacts have been observed in other species. For example, compressed and bruised eggs have been found in *Macquaria ambigua* and *Macquaria novemaculeata* after retrieval from >*~*10 m [15,16].

Irrespective of any physical gonad damage caused by barotrauma, other studies showed that angling fish results in stress responses which may lead to physiological changes [14,17−20], affecting subsequent gonadal development. For example, studies on line-caught *C*. *auratus* [14,21,22] and *M*. *novemaculeata* [23], revealed that gonad condition is related to stress, and ovarian development might stall [18,20,23,24] or even fail completely [24,25].

Despite the above, no published studies have specifically tested hypotheses associated with barotrauma impacts among reproductively active *C*. *auratus*. As a step towards investigating this shortfall, our first aim was to quantify the immediate gross physical impacts of barotrauma on *C*. *auratus* and their gonads after being angled from typical depths (8–70 m) during their full reproductive development. A second aim was to investigate the importance of technical (e.g. angler, hooking location, playing time and time landed), environmental (e.g. fishing depth, location and month), and biological (e.g. sex, TL, reproductive stage and stomach contents) factors in explaining variability among the observed impacts of barotrauma.

## Materials and Methods

### Ethics statement

Approval was granted by the NSW Marine Parks Authority (permit number 2011/003) for fish to be angled within the Solitary Islands Marine Park, New South Wales (NSW), Australia. Animal ethics approval was issued by the NSW DPI Animal Care and Ethics Committee (Reference 03/12). None of the work involved threatened, endangered or protected species.

### Study location

Fish were collected offshore within the Solitary Islands Marine Park between Wooli (29°56’S 153°26’E) and Nambucca Heads (30°34’S 153°13’E), NSW, Australia between June and October 2011. During 10–12 days each month, up to 30 anglers used rods-and-reels fitted with commonly used soft plastic lures or natural baits to target *C*. *auratus* [3,6,7] which were then assessed by researchers. Sea surface water temperatures were recorded at many, but not all fishing locations.

The data collected for all angled fish included: their depth of capture; Julian calendar day; time landed; general hook location (i.e. mouth, ingested or body); play time (s); TL (to the nearest mm); and the presence or absence of external haemorrhaging (e.g. blood at the hook wound), oocytes or sperm (milt) expelled around the vent, oculogyration reflex (movement of the eye about the antero-posterior axis), gastric herniation (into the buccal cavity or out of the mouth), exophthalmia, corneal or subcutaneous gas bubbles, or a prolapsed cloaca. Wherever feasible, the external clinical signs of barotrauma were observed and photographed immediately after capture for comparative reference and while the fish was alive (all within 1 min of capture). Each fish was then euthanized (in an ice slurry) before being transferred to the laboratory to be dissected and clinically examined for internal signs of barotrauma, including the presence or absence of: haemorrhages in the liver, kidneys or coelomic cavity; organ displacement; and a ruptured swim bladder. If present, the length of the swim bladder rupture was measured to the nearest 0.1 mm.

For each fish, the entire digestive tract was excised and ranked as full or empty, while the gonads were macroscopically assessed for haemorrhages, ruptures or oedema. The subsequent appearance of the gonads was used to sex each fish and determine their reproductive development following the five stages described by Wakefield et al. [1]: I-immature or resting, II-developing, III-developed, IV-ripe or spawning, and V-spent.

### Data analyses

The fixed continuous and categorical effects of fish ‘TL’, ‘sex’, ‘play time’,‘reproductive stage’, ‘capture depth’, ‘digestive tract contents’ and ‘Julian date’ (i.e. defined as days subsequent to 8 June, 2011) and any interactions were included along with the random terms of ‘anglers’, ‘fishing locations’ and ‘months’ in mixed-effects logistic models fitted to explain variability among the observed (1) clinical signs of barotrauma and (2) barotrauma-induced impacts to gonads. For each analysis, a stepwise variable search algorithm was employed with the most parsimonious model based on the lowest Akaike’s Information Criterion (AIC). Significant log odds for categorical variables were centred for confounding effects (of other terms) and subsequently explored using the Benjamini-Hochberg-Yekutieli procedure to control the false discovery rate (FDR; [26]). All fits were obtained using the lmer function in the lme4 package of the freely available R language [27].

## Results

In total, 90 female (mean TL ± SD of 48.92 ± 13.10 cm) and 90 male (48.71 ± 11.74 cm TL) *C*. *auratus* were caught from 8.0–63.7 m (mean ± SD of 25.4 ± 9.7 m) over 22 fishing days (with a mean ± SD of 8.18 ± 7.07 fish per day). All fish were alive and moving vigorously during landing. Most fish were mouth hooked (92%), with fewer either ingesting the hook into their oesophagus (4%) or stomach (2%) or were externally body hooked (2%). For those fish where the terminal rig and/or bait type was recorded (*n* = 122), 48% were angled using lures, while 52% were caught using baited hooks with *Sardinops sagax* (Jenyns 1842) (nearly all of the 52%), although *Sarda* sp. (Cuvier 1829), *Loligo* sp. (Lamar 1798), *Scomber australasicus* (Cuvier 1832) and *Auxis thazard thazard* (Lacepède 1800) were also used. All *C*. *auratus* were played for between 10 and 360 s (mean ± SD of 77.3 ± 76.3 s). Surface water temperatures remained similar within and among days, with an overall mean (± SD) of 19.9 ± 0.6°C.

### Fish condition

Females and males were similarly represented within (but less so among) reproductive stages with immature or resting (34.4 and 21.1%, respectively of all fish), ripe or spawning (20.0 and 25.6%) and spent (32.2 and 35.6%) individuals more dominant than those that were either developing (6.7 and 10.0%) or developed (6.7 and 7.8%). All fish displayed oculogyration reflex. Table 1 shows the occurrence of the different signs of barotrauma found in all fish. In terms of the clinical signs of barotrauma (and irrespective of their sex) no *C*. *auratus* had any corneal or subcutaneous gas bubbles, or exophthalmia, and only two females and three males had haemorrhaging kidneys. Colemic cavity haemorrhaging was the next least common clinical sign of barotrauma (<36% for both sexes), while gastric herniation, external haemorrhaging and organ displacement was more common (40-55% of each sex). By comparison, 60-80% of both sexes exhibited a prolapsed cloaca, ruptured swim bladder and/or liver haemorrhaging (Table 1).

**Table 1:** Percentage occurrence of dichotomous internal and external clinical signs of barotrauma and impacts to gonads observed for 90 female (mean TL ± SD of 48.92 ± 13.10 cm) and 90 male (mean TL ± SD of 48.71 ± 11.74 cm) *Chrysophrys auratus* (and both sexes combined) angled from 8.0-63.7 m off New South Wales during four months from 8 June 2011.

			Females	Males	Combined
Clinical signs of barotrauma					
	*External*				
		Corneal gas bubbles	0.0	0.0	0.0
		Subcutaneous gas bubbles	0.0	0.0	0.0
		Exophthalmia	0.0	0.0	0.0
		Prolapsed cloaca	61.1	58.9	60.0
		Gastric herniation	43.3	47.8	45.6
		External haemorrhaging	51.1	40.0	45.6
	*Internal*				
		Ruptured swim bladder	68.9	77.8	73.3
		Kidney haemorrhaging	2.2	3.33	2.8
		Liver haemorrhaging	66.7	80.0	73.3
		Coloemic cavity haemorrhaging	35.6	31.1	33.3
		Organ displacement	43.3	53.3	48.3
Gonad impacts					
	Rupture		0.0	0.0	0.0
	Oedema		0.0	0.0	0.0
	Expelled milt or oocytes		0.0	0.0	0.0
	Haemorrhaging		12.2	17.8	15.0

**Fig 1:**
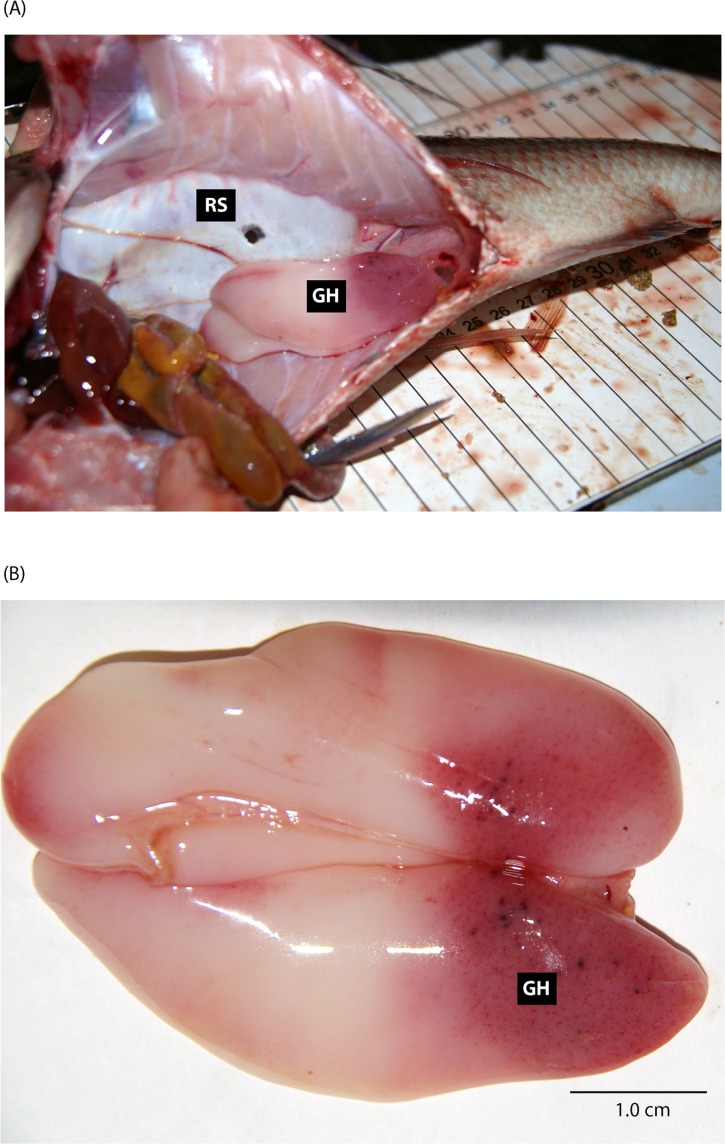
Internal view of the (A) coelomic cavity and (B) gonad of a male *Chrysophrys auratus* (38.5 cm TL) that was angled from 32.5 m and had a ruptured swim bladder (RS—1.3 cm) and posterior gonad haemorrhaging (GH). The location of the gonad haemorrhage was typical across all fish with this clinical sign. This fish also had a prolapsed cloaca, organ displacement and liver, kidney and coelomic cavity haemorrhages.

There were no observable ruptures, oedema or oocytes or milt protruding from vents. However, 12.2 and 17.8% of males and females presented evidence of gonad haemorrhaging (Table 1). While small sections of haemorrhaging were visible across the entire gonad in those afflicted fish, most was primarily located on the posterior end towards the anus (Fig. 1a, b).

### Analyses of clinical signs of barotrauma

For five of the informative clinical signs describing barotrauma, the fixed effects in the parsimonious logistic model (i.e. with the lowest AIC) were reduced to reproductive stage and capture depth (Table 2). The four variables not explained by this model were the (1) total number of clinical signs of barotrauma (with the model also comprising Julian date), (2) prolapsed cloaca (also comprising digestive tract content), (3) external haemorrhaging (only comprising Julian date, and both as a linear and quadratic term) and, where present, (4) the size of the swim bladder rupture (only comprising TL) (Table 2).

**Table 2:** Significance of log odds from parsimonious logistic models (with Akaike’s Information Criterion−AIC) assessing the importance of various fixed effects in explaining variability among the occurrence of clinical signs of barotrauma (and the total number) and gonad haemorrhaging in *Chrysophrys auratus* angled from 8.0-63.7 m off New South Wales during four months from 8 June 2011.

	Total no. of	Prolapsed	Gastric	External	Ruptured	Length of swim	Liver	Colemic cavity	Organ	Gonad
	clinical signs	cloaca	herniation	haemorrhaging	swim bladder	bladder rupture	haemorrhaging	haemorrhaging	displacement	haemorrhaging
Capture depth	***	***	***	Na	***	Na	***	***	***	−
Reproductive stage	***	***	***	Na	***	Na	**	***	*	***
Digestive tract contents	Na	*	Na	Na	Na	Na	Na	Na	Na	Na
Total length	Na	Na	Na	Na	Na	***	Na	Na	Na	Na
Julian date										
Linear	***	Na	Na	**	Na	Na	Na	Na	Na	●
Quadratic	Na	Na	Na	**	Na	Na	Na	Na	Na	Na
Males	Na	Na	Na	Na	Na	Na	Na	Na	Na	**
Julian date (linear) × males	Na	Na	Na	Na	Na	Na	Na	Na	Na	**
AIC	654	193.7	223.6	212.7	106.8	486.3	71.9	183.6	229.6	129.6

Significance is represented by:

−p>0.1,

●p<0.1,

*p<0.05,

**p<0.01,

***p<0.001,

Na = term not considered in the parsimonious model.

**Fig 2:**
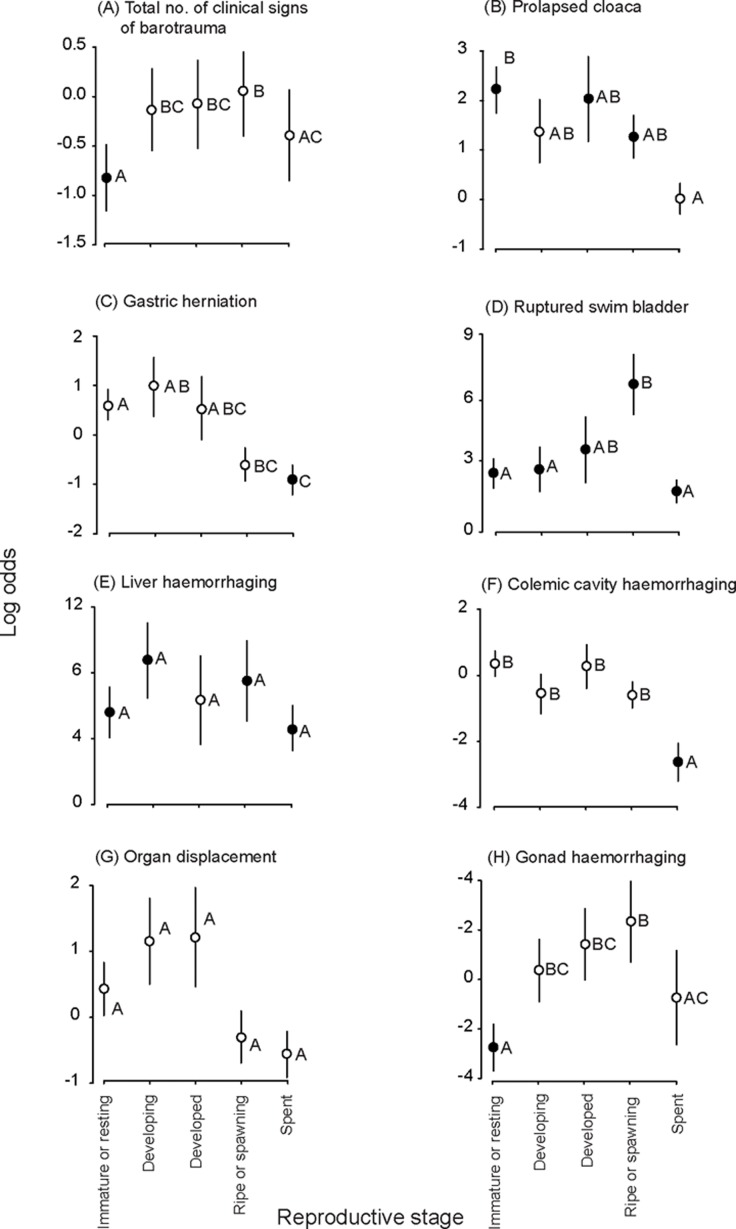
Log odds (± SE; standardized to mean depth) from logistic models across five reproductive stages of *Chrysophrys auratus* for (A) the total number of clinical signs of barotrauma, (B) prolapsed cloaca, (C) gastric herniation, (D) ruptured swim bladder, (E) liver haemorrhaging, (F) coelomic cavity haemorrhaging, (G) organ torsion and (H) gonad haemorrhaging. The significance of the log odds is designated by either black (*p*<0.05) or white (*p>*0.05) circles, while the dissimilar letters represent differences detected in false-discovery-rate pairwise comparisons (*p*<0.05).

Capture depth and reproductive stage were significant in all relevant parsimonious models, with capture depth always manifesting as a positive association with the probability of the clinical sign (LMM, *p*<0.05; Table 2). For reproductive stage, the subsequent log odds of occurrence (standardised to a mean depth of 25.3 ± 9.7 m) varied considerably, but generally fish that were either immature or resting, and especially developing, developed, or ripe or spawning had greater log odds for the response variables than those that were spent (Fig. 2a-g). Specifically, compared to spent fish, the log odds were significantly greater among (1) immature or resting individuals for a prolapsed cloaca, (2) immature or resting, and developing individuals for gastric herniation and (3) immature or resting, developing, developed, or ripe or spawning individuals for colemic cavity haemorrhaging (FDR, *p*<0.05; Fig. 2b, c, f). The clear exceptions to the above trend were the presence of a ruptured swim bladder and the total number of clinical signs of barotrauma, which mostly had greater log odds among ripe or spawning fish than the other four reproductive stages (Fig. 2a, d).

For the remaining continuous variables in the LMMs describing the clinical signs of barotrauma, Julian date was positively associated with (1) the total number of clinical signs and (2) external haemorrhaging (at a decreasing temporal rate), while the size of the swim bladder rupture (where present) was positively associated with TL (*p* <0.001; Table 2). For a prolapsed cloaca, there was also a significant increase in the log odds associated with food in the digestive tract (LMM, *p*<0.05; Table 2).

### Analyses of gonad impacts

The parsimonious model for gonad haemorrhaging (the only response variable describing gonad damage; Table 1) among fish comprised the fixed effects of reproductive stage and an interaction between sex and Julian date (*p*<0.01; Table 2). Irrespective of sex, immature or resting and spent fish had the lowest log odds for haemorrhaging (Fig. 2H), while among all fish the probability of haemorrhaging decreased with Julian date and at a significantly faster rate in males than females (*p*<0.01; Table 2).

## Discussion

The clinical signs of barotrauma and their severity among *C*. *auratus* in this study were comparable to those observed during previous studies on this species [7,8,27,28] and other teleosts angled from similar depths [9,15,29,30]. In the absence of ancillary deleterious factors such as hook ingestion or protracted air exposure, the extent of observed barotrauma has been associated with few immediate and short-term mortalities [7,8]. Assuming a similar response by *C*. *auratus* in this study, many might have been expected to survive surface release over the short term. Perhaps less clear, are the longer-term impacts, both in terms of mortality and sublethal consequences. The potential for such longer-term impacts can be discussed by first considering the observed morphological changes and then their apparent relationship with reproductive stage.

The most common clinical signs of barotrauma in *C*. *auratus* observed here were a liver haemorrhage, prolapsed cloaca and ruptured swim bladder. Of these injuries, liver haemorrhage was perhaps the least severe in terms of impacting welfare [9], although hepatic dysfunction is one possible outcome, which could result in eventual fatalities. Similarly, a prolapsed cloaca and ruptured swim bladder could lead to detrimental bacterial infections and in some cases, possibly septicaemia with associated mortality [31]. Further, beyond organ displacement and possible disruptions to blood supply, overexpansion of the swim bladder can cause ongoing buoyancy problems that could impair normal swimming behaviour, rendering fish less efficient at feeding and more susceptible to predation and exposure to sunlight [9,32].

The frequencies of the recorded clinical signs (and several others) were positively correlated with capture depth, which concurs with studies done on other species (e.g. *Glaucosoma hebraicum* [33]; *Tautoga onitis* [34]; and Sebastes spp [35]). For example, Rummer and Bennett [9] showed that the frequency of injuries to *Lutjanus campechanus* increased with pressure (depth) and also changed somewhat. Specifically, trauma injuries ranged from initial organ displacement-related impacts such as those observed here, to more severe compaction injuries as swim bladder expansion within the confines of the body wall, applying greater pressure on other coelomic-cavity organs [9].

Notwithstanding the above, the earlier study by Butcher et al. [7] assessing the fate of *C*. *auratus* angled from comparable depths to those here, did not show a similar correlation (i.e. there was no effect of depth). However, one key difference between the current study and that done by Butcher et al. [7] was the temporal sampling and associated reproductive condition of fish. Butcher et al. [7] targeted *C*. *auratus* between November and January, when they were predominantly stage I resting. Therefore, it is possible that the condition of fish at the time of capture strongly influences the observable signs of barotrauma and subsequent potential for mortality.

Immature or resting fish were generally less susceptible to variations across different depths and, along with spent fish, incurred relatively fewer overall impacts (irrespective of depth). From a morphological perspective, some of the observed differences might be explained by the physical effects of gonad development on available space in the coelomic cavity and the changes in volume of the swim bladder when fish are exposed to rapid decompression [9]. For example, irrespective of sex, compared to resting and spent fish, those that were developed and spawning would have less space for expanding organs in their body cavity and therefore more likely to present pressure-related injuries. Hall et al. [16] observed similar effects among stage IV female *M*. *ambigua* angled from deep water. This hypothesis of a correlation between pressure-related injuries and body cavity space is supported here by the (1) significant effect of food in the digestive tract (which may have restricted available space) and (2) negative relationship between Julian date and the frequency of clinical signs of barotrauma. More specifically, most spent fish were caught towards the end of sampling and therefore had more body-cavity space, especially among males.

The implications of macroscopic clinical signs of barotrauma on reproduction were limited to haemorrhaged gonads in developing, developed and ripe or spawning fish. Like the damage to swim bladders, the observed consistent gonad haemorrhaging possibly was caused by pressure-related changes and the subsequent displacement of organs [7,9]. Further, most of the haemorrhaging was confined to the posterior ventral surfaces of the gonad and probably occurred as the expanding swim bladder (and other organs) forced the gonads into the walls of the colemic cavity. Assuming such a mechanism, a related concern among spawning fish would be the premature extrusion of milt or oocytes [15,16], which although not observed here conceivably could reduce reproductive output.

It is also important to consider that regardless of the observed macroscopic injuries caused by barotrauma, there are likely to be more subtle cumulative physiological impacts of angling and handling *C*. *auratus* [17,14,20]. For example, *C*. *auratus* caught during their reproductive season may not only suffer common angling-related stressors such exhaustion and air exposure leading to physiological changes [17] or decreases in the concentration gonadal steroids [14,18,20] but for females also a rapid development of atretic (resorbing) ovarian follicles [23]. The latter effect could impact females throughout the spawning season [14,15,20] and be irreversible over the short-term [18]. Conceivably, pressure-related impacts and subsequent gonad haemorrhaging, as well as likely premature milt or egg extrusion [15,16], combined with stress-related negative responses, could have a cumulative impact; not only on the immediate health and condition of *C*. *auratus* but also on their short- and long-term reproductive success.

The results from this study can be considered a first step towards identifying the potential negative impacts of angling-induced barotrauma among *C*. *auratus* during their reproduction. Clearly, further studies are required to focus on the potential for (1) interactions between reproductive stage and depth, (2) space constraints in the coelomic cavity(and any sexual dimorphism), and (3) more detailed physiological consequences of barotrauma on gonads. The cumulative effects of gonad damage and other stress-related impacts on reproduction, and the timing and severity of angling in relation to gonadal development are crucial precursors for coherent management. Notwithstanding such shortfalls, given the predicted spawning locations and observed influences of depth and season on the severity of barotrauma and potential impacts among *C*. *auratus* off south eastern Australia, any identified negative consequences for reproduction might be managed simply by regulating either temporal or spatial effort.
